# Supplementary material for: Connecting higher education to workplace activities and earnings
Source: PLoS One. 2023 Mar 15;18(3):e0282323. doi: 10.1371/journal.pone.0282323 (PMC10016720; doi:10.1371/journal.pone.0282323)
Supplement: S1 File — (PDF) [file pone.0282323.s001.pdf]

Supplementary Information for

Connecting Higher Education to Workplace Activities and Earnings

Hung Chau, Sarah H. Bana, Baptiste Bouvier and Morgan R. Frank

Morgan R. Frank.
E-mail: mrfrank.pitt.edu

This PDF file includes:

Supplementary text
Figs. S1 to S13
Tables S1 to S2
SI References

Supporting Information Text

1. Section: OSP Data Processing

The OSP dataset contains roughly 3 million syllabi in the US. Each syllabus has multiple attributes. These include: a unique syllabus ID, the probability it is a syllabus (this is due to the automated process with which syllabi were originally scraped), the year, the FOS (and corresponding level of certainty of being that FOS), the institution, the location of the institution (latitude/longitude), the language, as well as metadata on the process (e.g., method used to collect the syllabus info). Our analysis only used a small subset of these, hence the potential for significant further study.

A. The Identification of Course Descriptions. There is multi-stage process to identify course descriptions from the raw syllabus data. To begin, the text associated with each syllabus includes multiple elements. Some are useful (e.g., course objectives, course descriptions, outline of the class), and others are less so (e.g., office hour times, contact details, administrative information). The first task is to keep the former (i.e., the elements that contribute to an understanding of the content taught in the class), and to exclude the latter.

Each syllabus text in the dataset is structured with multiple headings, including "Overview", "About the course", "Course content", "Description", "Course outline", "Outcome", "Objective", "Aim", and "Goal". We begin by splitting the syllabus text into these groups (using roughly a dozen 'useful' headings), so that we end up with text under each such heading. After some processing (e.g., removing duplicate text and pieces of text that are too similar: we use *SequenceMatcher* from the *difflib* (Python) library and do not include text that has a similarity > 0.8 with another already-added text). The concatenation of these groups forms the "course description" of the syllabus. Note, this method is imperfect due to the noise and complication of the format texts in OSP: there is text that is not being captured and likely small amounts of irrelevant administrative information included. However, our hypothesis is that the administrative text (e.g., "Office hours are between 3-5pm on Thursdays") is 'neutral' and does not influence the DWA analysis that follows. There is clearly scope for improvement with this process with more advanced natural language processing techniques, but we do believe the outcome of this process is efficient for our following analyses and do not cause misleading results.

B. The Language Embeddings to Compute DWA Syllabus Similarity. Once the text has been cleaned, the next process is to generate a vector to represent each DWA, using a Wikipedia-trained word embeddings (*fasttext-wiki-news-subwords-300*) in Gensim language models[*].

As the first step, we tokenize each DWA in the full list of 2070 DWAs. This tokenized list is used to create a (*genism.corpora*) dictionary. Then, we take that dictionary of DWAs to run bag-of-words (BOWs) on the tokenized syllabus text. We also generate the (*genism.models*) 'word embedding similarity index' from the pre-trained embeddings we used (trained on Wikipedia pages). Then, we create a sparse term similarity matrix between the language model similarity index and the dictionary based off of DWAs. We generate one final index by taking the *soft cosine similarity* between that similarity matrix and the bag-of-words representation of DWAs.

Once the initial processing has occurred, we move to analyzing each syllabus text in turn. First, we take the processed syllabus text and tokenize it a BOWs representation (removing words like *days of the week*, *months*, and *common words* like "http", "hour", "assignment", "college", "university", "emails", etc.). Using the final generated similarity index, we calculate the *soft cosine similarities* (1) between the BOWs representations of the DWAs and the syllabus. As the result, each syllabus is represented as a vector of 2070 dimensions, showing how strongly each of 2070 DWAs is associated with the course description. With this representation of course syllabi, we now can easily compute the relationship between each pair of course syllabi, representations of FOS and universities and so on.

[*] https://github.com/RaRe-Technologies/gensim-data

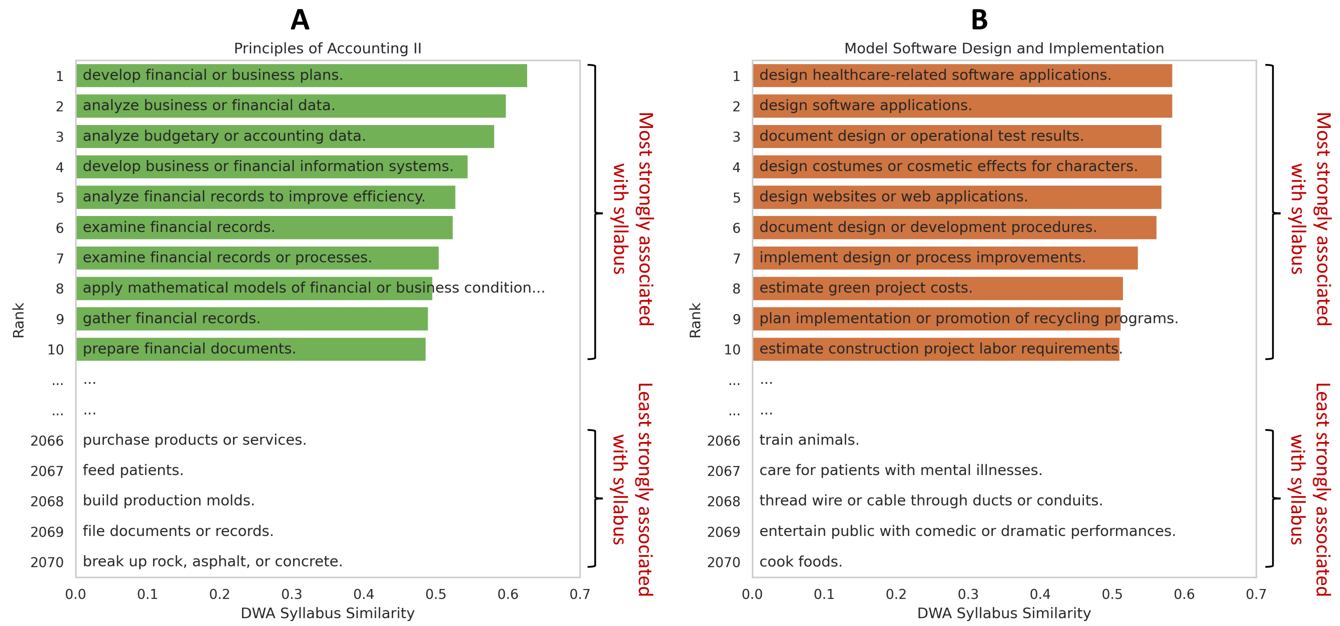

Fig. S1. (A) An example accounting syllabus and the activities that are most and least strongly associated with its course description; and (B) An example computer science syllabus and the activities that are most and least strongly associated with its course description. The course description and learning objectives are extracted and embedded into a pre-trained language space. DWA syllabus similarity scores (from *0* to *1*) are calculated for each detailed workplace activity against the syllabus.

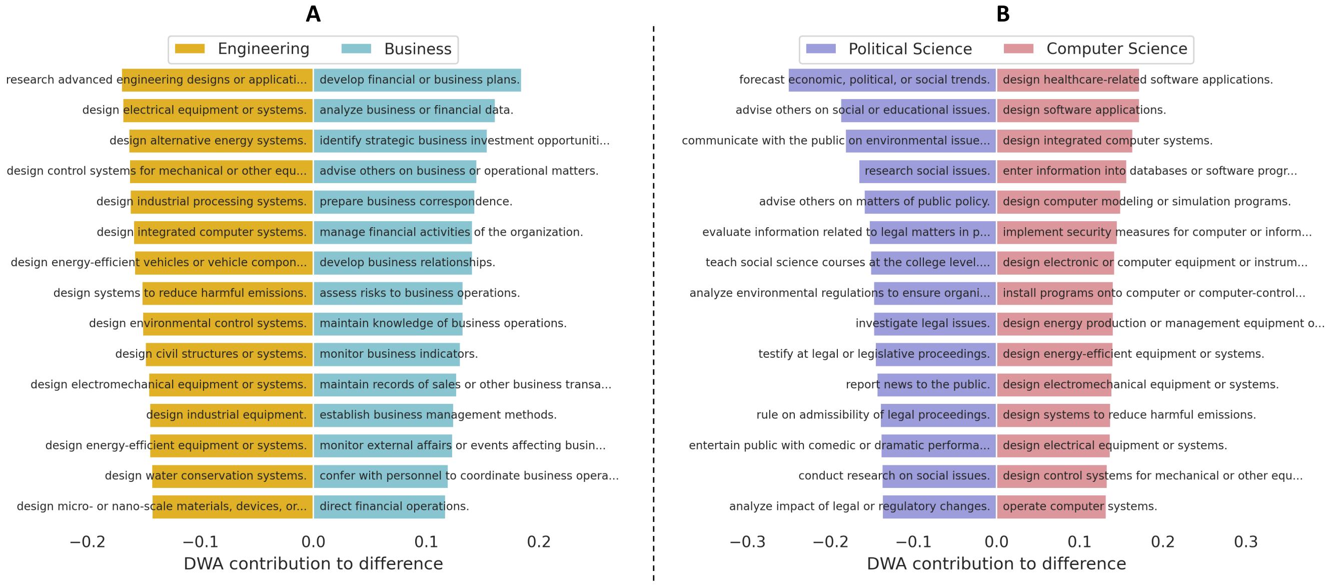

Fig. S2. (A) The DWAs that most significantly distinguish Engineering syllabi from Business syllabi. (B) The DWAs that most significantly distinguish Political Science syllabi from Computer Science syllabi.

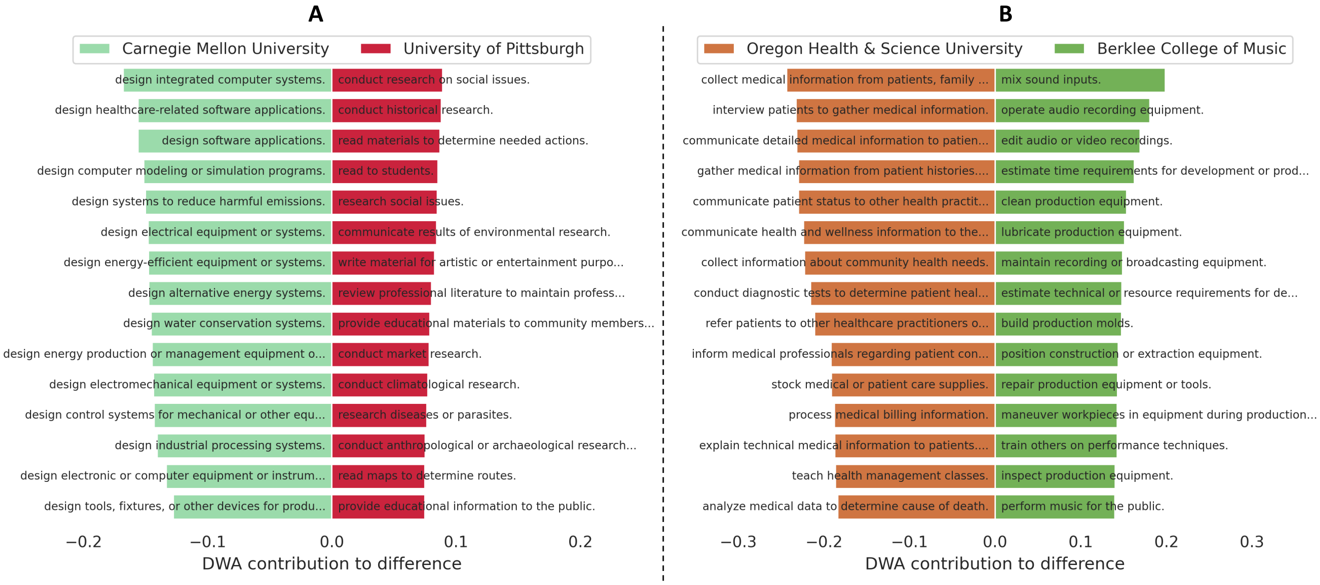

Fig. S3. (A) The DWAs that most strongly separate Carnegie Mellon University syllabi from University of Pittsburgh syllabi. (B) The DWAs that most strongly separate Oregon Health & Science University syllabi from Berklee College of Music syllabi.

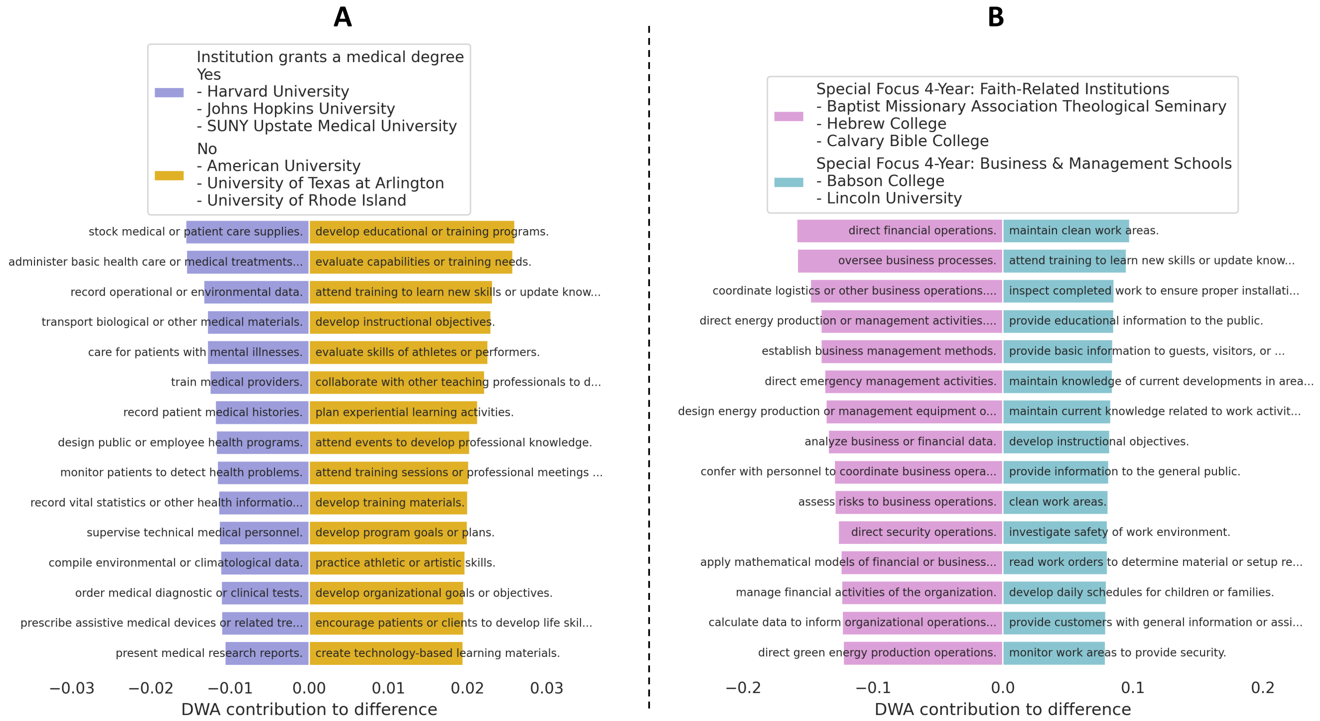

Fig. S4. (A) The DWAs that most strongly separate Medical Degree-Granting Schools syllabi from Non-Medical Degree-Granting Schools syllabi. (B) The DWAs that most strongly separate Special Focus 4-Year Faith-Related Schools syllabi from Business & Management Schools syllabi.

2. Distance Metric Correlation

To calculate DWA relationships, we experiment two different methods: (1) *Direct* - compute directly the cosine similarity of the embedding vectors; and (2) via course syllabi - based on the co-occurrence of dwa_1 and dwa_2 in course syllabi. For the second method, we experiment with four different similarity and distance metrics: *Cosine* similarity, *Euclidean* distance, *Manhattan* distance and *Jaccard* similarity. Figure S5 shows the correlations of these methods and distance metrics.

	Direct	Cosine	Euclidean	Manhattan	Jaccard
**Direct**	1.000000	0.520041	-0.302950	-0.278611	0.493206
**Cosine**	0.520041	1.000000	-0.214060	-0.225266	0.894886
**Euclidean**	-0.302950	-0.214060	1.000000	0.990004	-0.460567
**Manhattan**	-0.278611	-0.225266	0.990004	1.000000	-0.491437
**Jaccard**	0.493206	0.894886	-0.460567	-0.491437	1.000000

Fig. S5. The correlation matrix of the two methods and four distance metrics to calculate DWA relationships.

3. Predicting Educational Trends

A. Comparing Distance Metrics. For robustness checks, we run Models 2, 3, 4 & 5 (explained in the main manuscript) with the two different methods and four distance metrics for computing the DWA relationships. The first method (called *Direct)* computes the DWA relationships by directly measuring Cosine similarity of their language embedding vectors. This approach measures a static relationship between DWAs and can not distinguish the dynamics of how one DWA relates to another locally (i.e., within a FOS or a university) and globally (i.e., across all of academia). The second method calculates the relationship between each pair of DWAs based on the co-occurrence of the two DWAs in course syllabi. The relationships can be measured locally as well as globally. We experiment four different distance metrics for the second method: Cosine similarity (*Cosine*), Euclidean distance (*Euclidean*), Manhattan distance (*Manhattan*) and Jaccard similarity (*Jaccard*).

Figure S6 and S7 show the RMSE and R-Squared performance comparisons of these methods and distance metrics for each of the models involving inter-DWA relationships. As can be seen from the figures, *Jaccard* performs best consistently across all the models. Second from the best is *Cosine* similarity metric. *Direct* method fails to distinguish the dynamics of how one DWA relates to another locally and globally; as the results, for the best model (i.e., Model 5), it performs worst among all the variations. On the other hand, the global relationships captured by *Manhattan* and *Euclidean* help them surpass *Direct* performances.

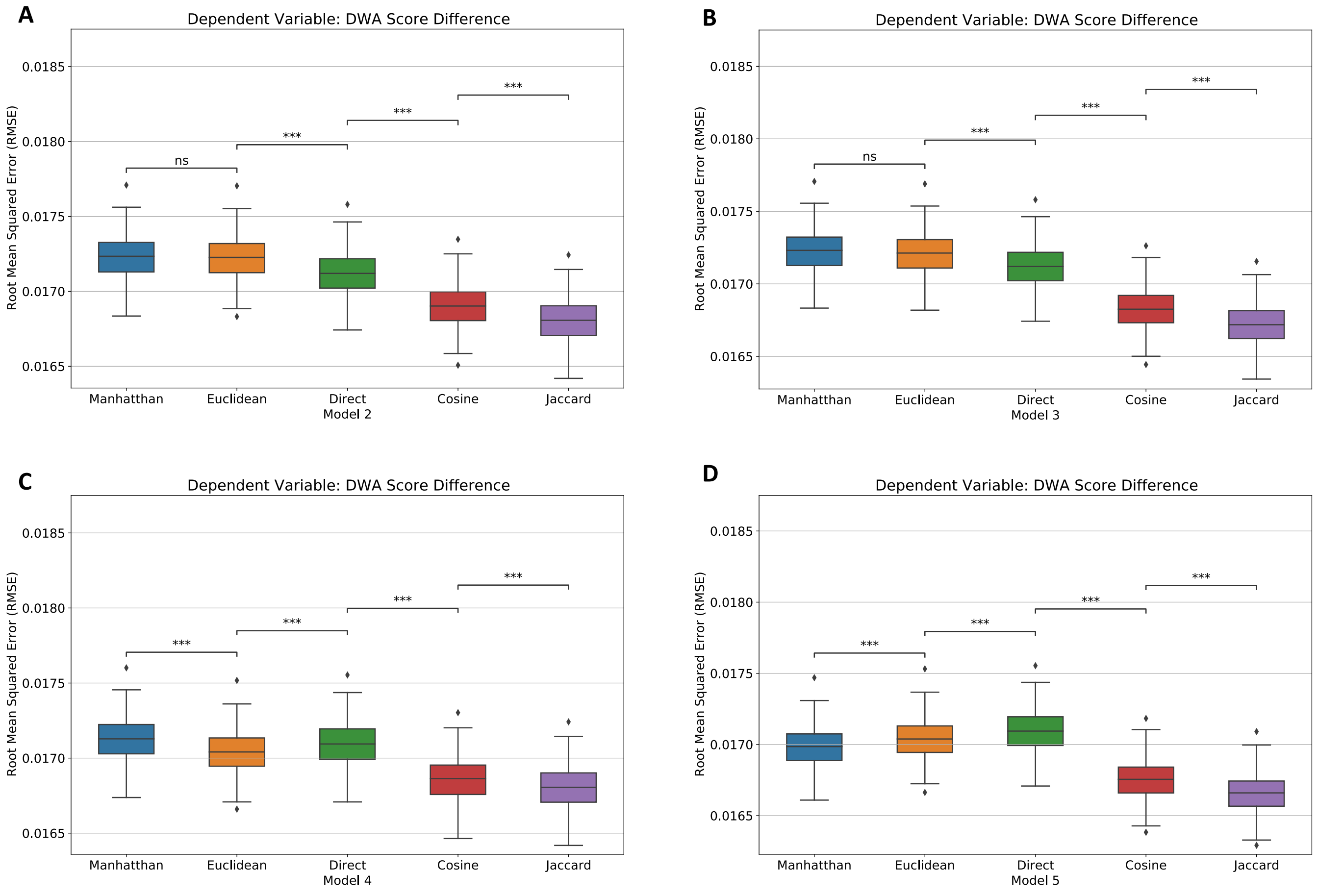

Fig. S6. Workplace activities detected from syllabi predicting teaching dynamics within a field of study and earnings of college graduates. We perform 5-fold cross validation and repeat 40 times (i.e., 200 trials in total) for each model and measure RMSE by the resulting models applied to the test set. Asterisks indicate the statistically significant difference between two models' performances with Bonferroni correction. (A) Predicting the importance of DWAs changing in 10 years (2008 vs. 2017). (A), (B), (C) and (D) show the performance comparisons of different distance metrics calculating DWA relationships for Model 2, 3, 4 and 5, respectively.

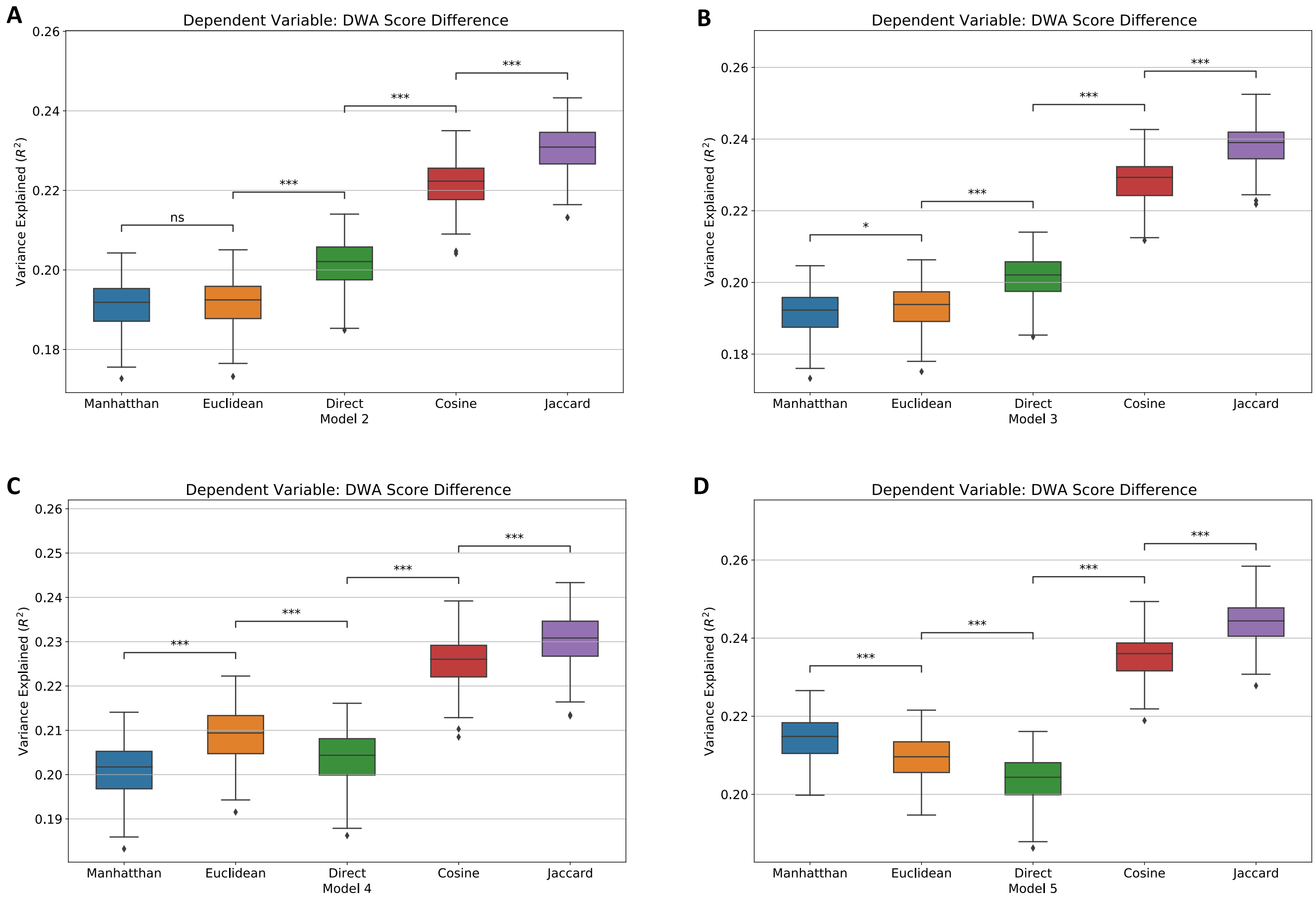

Fig. S7. Workplace activities detected from syllabi predicting teaching dynamics within a field of study and earnings of college graduates. We perform 5-fold cross validation and repeat 40 times (i.e., 200 trials in total) for each model and measure the variance explained (i.e., R^2) by the resulting models applied to the test set. Asterisks indicate the statistically significant difference between two models' performances with Bonferroni correction. (A) Predicting the importance of DWAs changing in 10 years (2008 vs. 2017). (A), (B), (C) and (D) show the performance comparisons of different distance metrics calculating DWA relationships for Model 2, 3, 4 and 5, respectively.

B. Classification Analysis. In additional to the regression analysis for educational trends presented in the main text, we perform a classification analysis for this problem. The task is to predict which DWAs become "important" in future, meaning those DWAs are not considered important at the current time but potentially are important in future (10 years later). This helps to understand how fields of study evolve overtime, enabling proactive course design by educators and informing educational incentives from policy makers.

"Important" DWAs are the DWAs that are the most prevalent ones for a FOS. DWA (a) is labeled as "important" in a FOS (f) when it satisfies the condition below:

$$r_f(dwa) >= \mu_f + 2 * \sigma_f \qquad [1]$$

Where μ_f and σ_f are the mean and the standard deviation of the relationships between the DWAs and the FOS, respectively. On average, there are around 59 and 58 "important" DWAs per FOS in 2008 and 2017, respectively. Number of DWAs that are important in 2017 but not important in 2008 is 15. These DWAs are positive labels in our classification analysis.

We build a logistic regression model to classify whether a DWA is important (1) or not (0). We use the information about the current propensity score of the DWA and its relationships with currently important DWAs calculated with Jaccard similarity metric. Based on the principle of relatedness (2), our assumption is that skills co-taught with currently important skills are likely to become more important in future. To evaluate how well the model performs, we report the ROC curves and AUC scores for each individual FOS (see Figure S8). Since there is an unbalance in numbers of data points in the two classes (0 vs. 1), we, in addition, measure the model performance in terms of *precision*, *recall* and *F1-score* at top N (see Figure S9).

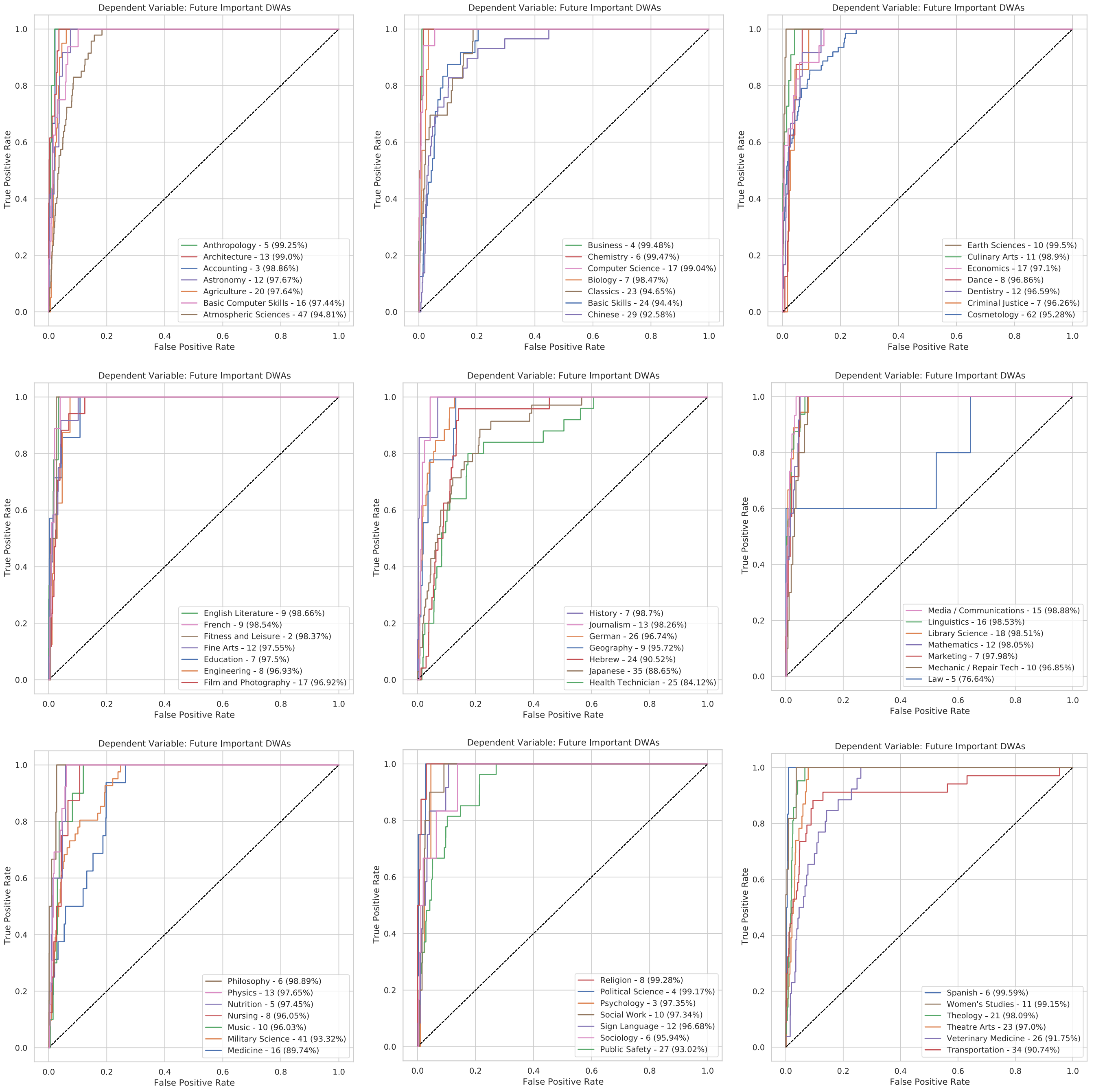

Fig. S8. ROC curves of the important-DWA classification model for each individual FOS. The legends display the field name, the numbers of important DWAs, and the AUC scores.

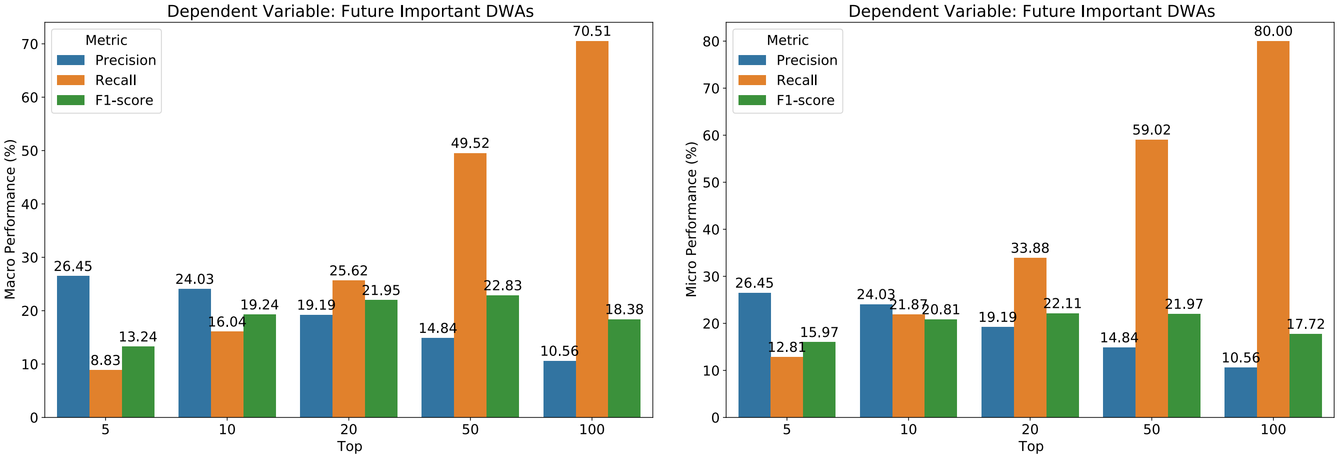

Fig. S9. Precision, recall and F-scores of the important-DWA classification model at top *N*. Macro performance is calculated when considering the prediction for all FOS together; while, micro performance is the average performance of each of individual FOS.

4. Selection of Graduate Earnings Records

College Scorecard Earnings provides transparency and consumer information related to individual institutions of higher education and individual fields of study within those institutions. We only process earnings records for Baccalaureate colleges and universities. We map College Scorecard CIP codes to OSP CIP codes. As a result, each earnings record includes the field name and institution information. There are 9007 graduate earnings records in 54 fields-of-study at 832 institutions.

To understand how workplace activities extracted from course syllabi contribute to earnings of graduates. We aggregate DWAs from the course syllabi taught at individual academic fields at specific institutions. Those DWAs are the features to predict graduate earnings presented in the main text. Though large, the OSP course syllabus data is not distributed evenly across fields-of-study and institutions. Some fields and institutions have much less course syllabi. Thus, to sufficiently estimate work activities taught in a FOS at a university, we limit earnings records for FOS (in an institution) that have at least 10 course syllabi. As a result, we obtain 2872 earnings records in 47 FOS at 347 institutions. Further more, we select FOS that have at least 30 earnings records across institutions for prediction tasks, resulting to the remaining 2601 earnings records in 26 FOS at 343 institutions (see Table S1 for details of numbers of observations of FOS in our analysis before and after filtering).

It is possible that a subset of earnings records of a FOS does not effectively represent the distribution of the entire population. We perform the Kolmogorov-Smirnov (KS) statistical test to make sure the remaining earnings records representative for the entire population of the field at the institute. If the remaining earnings observations passes the KS test ($p\text{-}value > 0.05$), we will keep that FOS for the earnings prediction analyses. As an example, Figure S10 plots the distribution of median earnings of graduates in *Business* against number of syllabi. Table S1 shows the *p-values* of the KS test for the FOS in our "*Within Field-of-Study Skill Variation and the Earnings of Recent College Graduates*" analysis in the main text.

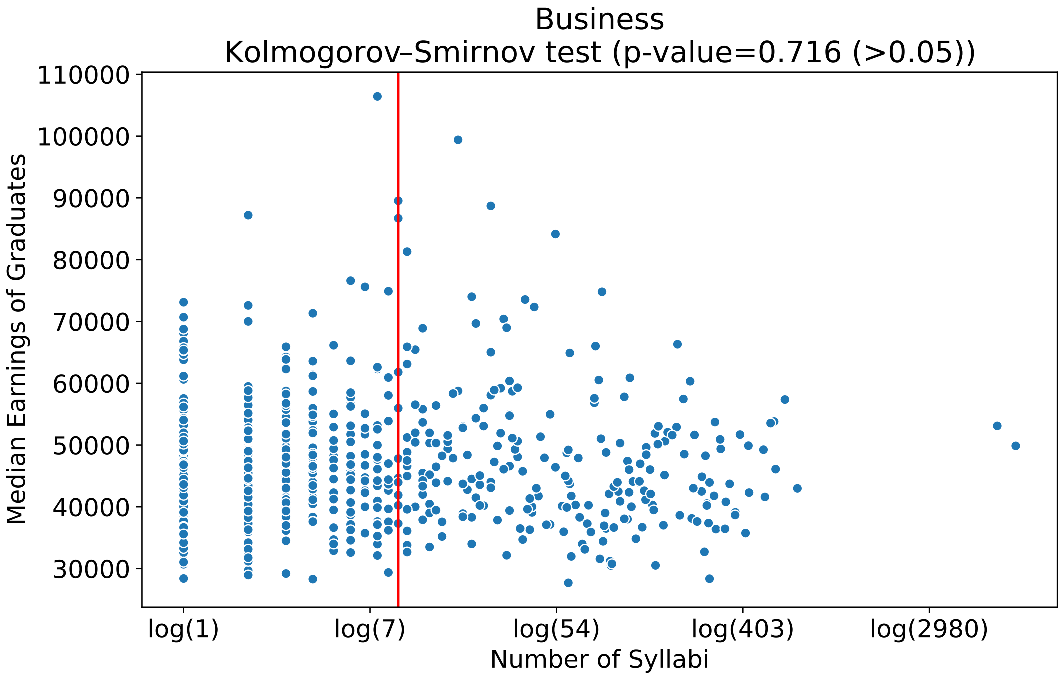

Fig. S10. Kolmogorov-Smirnov (KS) statistical test for the subset of median earnings of graduates in *Business*. The subset distribution passes the test with the *p-value* = 0.716 (>0.05). For the visualization purpose, we use the natural logarithm of number of the syllabi in the x-axis. The data points which are on the red line and the right of the red line belong to the selected subset used in our analysis.

Table S1. Numbers of earnings records of the top ten FOS that have passed the Kolmogorov–Smirnov test with the p-values < 0.05.

Field-of-Study	Number of records (after filtering)	Number of records (before filtering)	P-value (Kolmogorov–Smirnov test)
Business	246	683	0.716
Computer Science	198	640	0.06
Biology	181	563	0.89
Psychology	173	539	0.742
Mathematics	142	399	0.53
English Literature	135	526	0.81
Political Science	132	424	0.981
Education	122	424	0.419
Media / Communications	119	375	0.737
Accounting	109	224	0.542

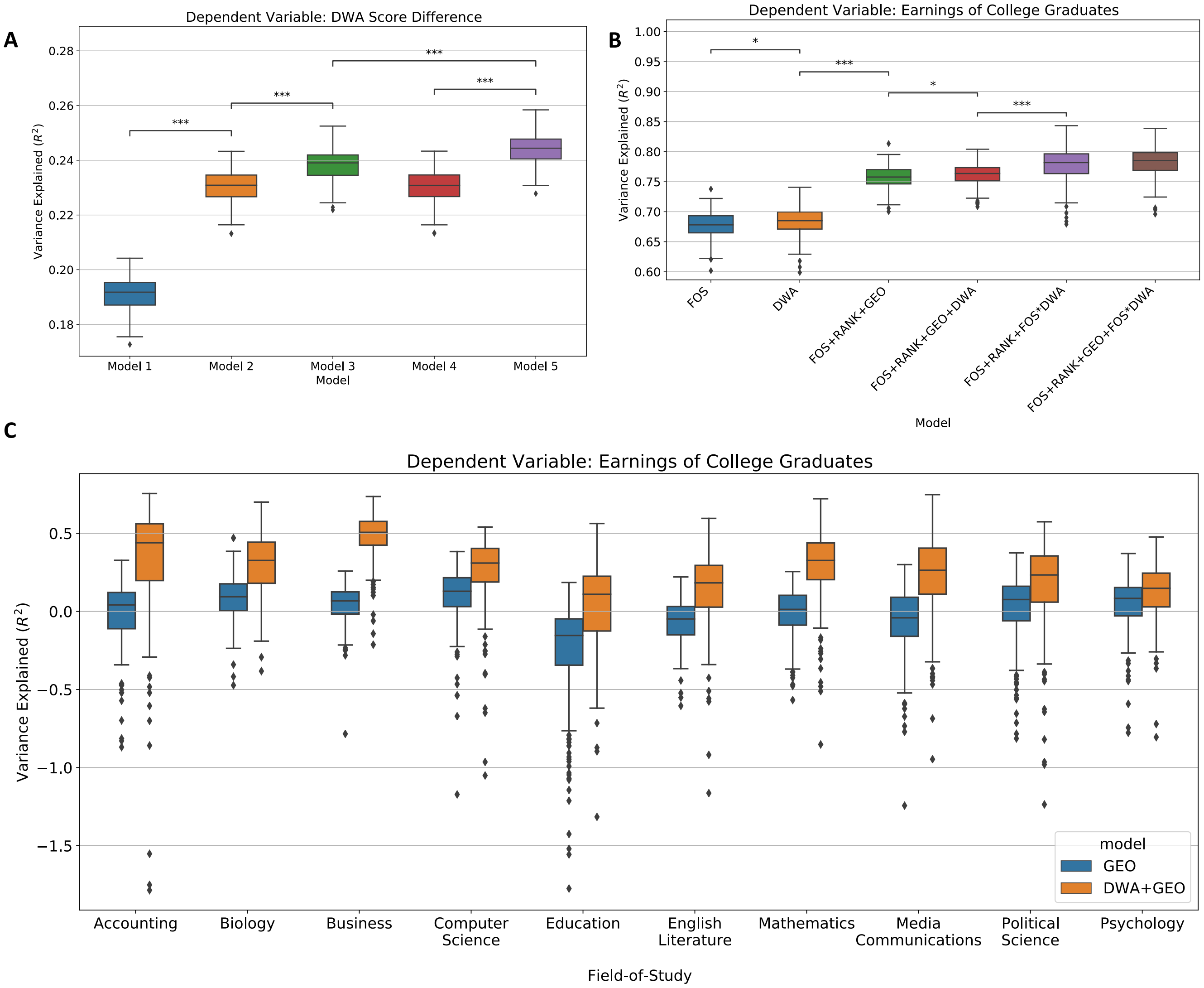

$p <= 0.05^{*}$, $p <= 0.01^{**}$, $p <= 0.001^{***}$

Fig. S11. Workplace activities detected from syllabi predicting teaching dynamics within a field of study and earnings of college graduates. We perform 5-fold cross validation and repeat 40 times (i.e., 200 trials in total) for each model and measure the variance explained (i.e., R^2) by the resulting model applied to the test set. Asterisks indicate the statistically significant difference between two models' performances with Bonferroni correction. (A) Predicting the importance of DWAs changing in 10 years (2008 vs. 2017). As a baseline, model 1 only considers the current DWA score and FOS fixed effects. The other models consider the relationships between DWAs calculated with Jaccard similarity, how they interact with each other to predict how they may change in future. (B) Predicting median earnings of graduates across all FOS. As a baseline, we consider the FOS and RANK fixed effects to predict earnings. (C) Predicting median earnings of graduates within FOS. The baseline model is the mean earnings of graduates of that FOS. The performances of the DWA models are statistically significantly better than the baseline models with the *p-values* < 0.001 for all of the reported FOS. $R^2 < 0$ happens in cross validation settings when the fitted model performs worse than the mean of the test set; especially, when the model is over-fitting and (or) suffers outlier issues.

Table S2. DWAs that have significant coefficients in the OLS regression analysis of the Earnings of Recent College Graduates.

Field-of-Study	Detailed Work Activity	Coefficient	P-value
Business	advise others on career or personal development.	1.647	0.00957
	complete documentation required by programs or regulations.	1.805	0.00238
	conduct health or safety training programs.	-2.674	0.00005
	direct criminal investigations.	-2.175	0.00546
	evaluate program effectiveness.	2.392	0.00002
	explain project details to the general public.	-1.62	0.01865
	explain use of products or services.	-1.702	0.0421
	position construction forms or molds.	2.434	0.04461
	research methods to improve food products.	1.514	0.01467
	review laws or regulations to maintain professional knowledge.	-1.25	0.04926
Computer Science	estimate labor or resource requirements for forestry, fishing, or agricultural operations.	-1.509	0.0367
	explain technical medical information to patients.	-1.536	0.0097
Biology	coordinate personnel recruitment activities.	-2.101	0.00095
	direct technical activities or operations.	-1.714	0.0174
	plant greenery to improve landscape appearance.	-2.294	0
	prepare outgoing mail.	1.898	0.04589
	test characteristics of materials or structures.	0.906	0.03263
Psychology	diagnose neural or psychological disorders.	0.635	0.04753
	distribute instructional or library materials.	1.213	0.03618
	evaluate patient functioning, capabilities, or health.	1.931	0.00049
	plan menu options.	1.621	0.01161
	refer clients to community or social service programs.	-0.973	0.01262
	select resources needed to accomplish tasks.	-1.44	0.03091
Mathematics	conduct diagnostic tests to determine patient health.	-0.869	0.04136
	schedule activities or facility use.	-0.965	0.03244
	teach online courses.	-1.019	0.02433
English Literature	adjust routes or speeds as necessary.	-1.504	0.021
	design energy production or management equipment or systems.	1.875	0.01623
Political Science	–	–	–
Education	design integrated computer systems.	1.024	0.02689
	teach social science courses at the college level.	0.526	0.02646
Media / Communications	confer with managers to make operational decisions.	1.911	0.00424
	review art or design materials.	1.137	0.02602
	serve on institutional or departmental committees.	1.751	0.00426
Accounting	advise others on career or personal development.	3.863	0
	develop artistic or design concepts for decoration, exhibition, or commercial purposes.	-1.803	0.00111
	make decisions in legal cases.	1.35	0.02197
	participate in staffing decisions.	1.664	0.03567
	process animal carcasses.	-1.113	0.02748
	promote educational institutions or programs.	-1.738	0.01258
	promote environmental sustainability or conservation initiatives.	-2.167	0.03087

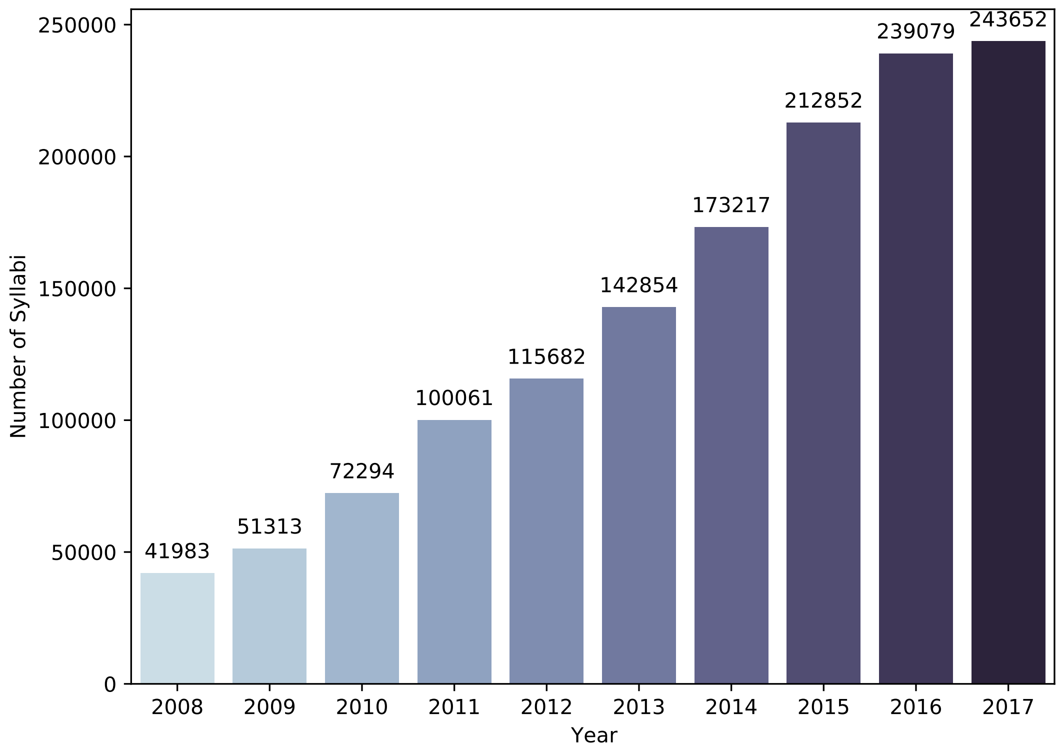

Fig. S12. Course statistics per year in OSP data.

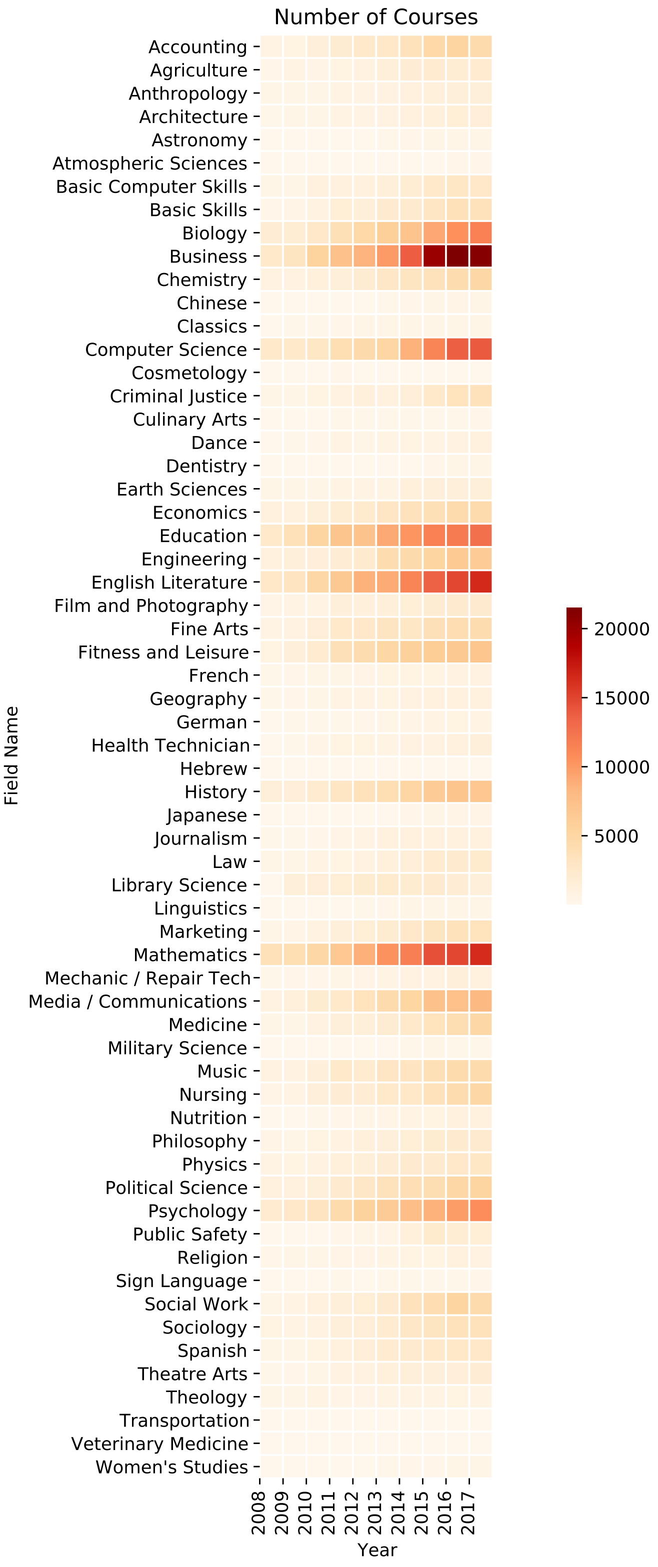

Fig. S13. Course statistics per year and per FOS in OSP data.

References

1. HGA Grigori Sidorov, Alexander Gelbukh, D Pinto, Soft similarity and soft cosine measure: Similarity of features in vector space model. *Comput. y Sistemas* **18**, 491–504 (2014).
2. CA Hidalgo, et al., The principle of relatedness in *Unifying Themes in Complex Systems IX*, eds. AJ Morales, C Gershenson, D Braha, AA Minai, Y Bar-Yam. (Springer International Publishing, Cham), pp. 451–457 (2018).
