# Supplementary material for: Connecting higher education to workplace activities and earnings
Source: PLoS One. 2023 Mar 15;18(3):e0282323. doi: 10.1371/journal.pone.0282323 (PMC10016720; doi:10.1371/journal.pone.0282323)
Supplement: Attachment — Submitted filename: Response to Reviewers.pdf [file pone.0282323.s003.pdf]

RESPONSE TO REVIEWERS

We thank the reviewers for their critical and extensive assessment of our work "**Connecting Higher Education to Workplace Activities and Earnings**" submitted for consideration for publication PLOS ONE - PONE-D-22-21083. In the following we address their concerns point by point. We also specify revisions to the main paper. We were able to address all of the reviewers' concerns and we feel like the manuscript is significantly improved.

REVIEWER COMMENTS

Reviewer #1 (Remarks to the Author):

The sections and subsections are not numbered as the authors claim in their responses.

Authors: We have numbered the sections in the manuscript as suggested.

The Materials and Methods Section (Lines 68 to 119) only shows materials (databases used). Actually, the methods appear starting from line 120 in the Results Section. Check this out.

Authors: We thank the reviewer for pointing it out. We have updated the section title as follows:

- "Materials and Methods" to "Materials"
- "Results" to "Methods and Results"

I keep seeing the term "propensity score" as confusing. It should be better clarified in the manuscript. Is it really the term that is used in computer science literature?

Authors: To reduce the confusion, we have changed "propensity score" to "relevance score" throughout the manuscript, and also added the definition of "relevance score" after the Equation (1) as follows "*These relevance scores are a measure of how strongly the skill (i.e., dwa) is represented in a field or university*".
